# Serum leptin and its relation to anthropometric measures of obesity in pre-diabetic Saudis

**DOI:** 10.1186/1475-2840-6-18

**Published:** 2007-07-07

**Authors:** Nasser M Al-Daghri, Omar S Al-Attas, Khalid Al-Rubeaan, Mehad Mohieldin, Mohammad Al-Katari, Alan F Jones, Sudhesh Kumar

**Affiliations:** 1 Biochemistry Department, College of Science, King Saud University, Riyadh, Saudi Arabia; 2 Biochemistry Department, College of Science, King Saud University, Riyadh, Saudi Arabia; 3 Medicine Department, College of Medicine, King Saud University, Riyadh, Saudi Arabia; 4 Diabetic Center, KAUH, Riyadh, Saudi Arabia; 5 Diabetic Center, KAUH, Riyadh, Saudi Arabia; 6 Clinical Biochemistry, Birmingham Heartlands Hospital, Birmingham, B9 5SS, UK; 7 Warwick Medical School, Diabetes & Metabolism Unit, University of Warwick, Coventry, CV4 7AL, UK

## Abstract

**Background:**

Little information is available on leptin concentrations in individuals with IGT. This study aims to determine and correlate leptin levels to anthropometric measures of obesity in pre-diabetic, (IFG and IGT), type 2 diabetic and normoglycaemic Saudis.

**Methods:**

308 adult Saudis (healthy controls n = 80; pre-diabetes n = 86; Type 2 diabetes n = 142) participated. Anthropometric parameters were measured and fasting blood samples taken. Serum insulin was analysed, using a solid phase enzyme amplified sensitivity immunoassay and also leptin concentrations, using radio-immunoassay. The remaining blood parameters were determined using standard laboratory procedures.

**Results:**

Leptin levels of diabetic and pre-diabetic men were higher than in normoglycaemic men (12.4 [3.2–72] vs 3.9 [0.8–20.0] ng/mL, (median [interquartile range], p = 0.0001). In females, leptin levels were significantly higher in pre-diabetic subjects (14.09 [2.8–44.4] ng/mL) than in normoglycaemic subjects (10.2 [0.25–34.8] ng/mL) (p = 0.046). After adjustment for BMI and gender, hip circumference was associated with log leptin (p = 0.006 with R2 = 0.086) among all subjects.

**Conclusion:**

Leptin is associated with measures of adiposity, hip circumference in particular, in the non-diabetic state among Saudi subjects. The higher leptin level among diabetics and pre-diabetics is not related to differences in anthropometric measures of obesity.

## Background

Saudi Arabia, a Middle Eastern country with a population of 22 million, has undergone significant economic and cultural changes over the past thirty years. Approximately 60% of the population are urbanised and have adopted a 'Westernised' lifestyle in terms of diet and physical activity [1]. Obesity and associated components of the metabolic syndrome have become increasingly common in the Saudi population, which may partially explain the increased incidence of coronary heart disease (CHD) [2-7]. In the USA, 17.1% of overweight adults aged 45–74 years were found to have impaired glucose tolerance (IGT); 11.9% had impaired fasting glucose (IFG); 22.6% had prediabetes; and 5.6% had both IGT and IFG [8].

Increased risk of CHD in subjects with IGT is supported by data from the Honolulu [8], Chicago [9] and Islington [10] heart studies, which demonstrated that mortality from CHD increased gradually as glucose tolerance deteriorated, with no apparent threshold value. In 1997, the American Diabetes Association (ADA) defined pre-diabetes as fasting plasma glucose (FPG) 6.1–7.0 mmol/L (110–126 mg/dl) [11]. The choice of the 6.1 mmol/L threshold was based partly on the increased risk of developing both microvascular and macrovascular complications above this level [11]. However, it has now been shown that the fasting glycaemia threshold of 6.1 mmol/L does not create a category of glucose homeostasis equivalent to that of IGT, either for the subsequent development of diabetes or CHD [12].

Diabetes increases the risk of developing cardiovascular disease by 2–3 times and increases by as much as 50% the risks of non-cardiovascular mortality associated with this condition [13-15]. This high risk is not completely explained by the traditional risk factors [16,17]. Pre-diabetes is also associated with cardiovascular diseases (CVD) [18,19], but it is unclear if it is an independent risk factor, because it commonly co-exists with other cardiovascular risk factors present in the metabolic syndrome. The overall prevalence of type 2 diabetes in Saudi adults is 23.7% [20] while the overall prevalence of CHD is 5.5% [5]. However, the percentage of Saudi subjects who had an FPG in the impaired fasting glucose range (6.1–7.0 mmol/L) was 14.1% [7].

In human beings, serum leptin concentration is directly proportional to body fat mass, but it is leptin resistance and not leptin deficiency *per se *which is regarded as a pathogenic mechanism in human obesity. Leptin concentrations vary widely among individuals with similar fat mass, indicating other possible factors for its determination [21,22]. Leptin may be a marker of risk of CHD, at least in males, and contributes to the CHD risk profile in subjects with insulin resistance [23]. Not much information is available on leptin concentrations in individuals with IGT and to our knowledge there has been no study of leptin in pre-diabetics within the region.

This study aims to determine and compare the serum leptin concentrations of diabetic and non-diabetic patients with those in patients who have pre-diabetes and to correlate it with anthropometric measures of obesity.

## Methods

### Subjects

In the present study, subjects were selected from the roster of adult, ambulant, non-pregnant patients attending the Diabetes Center at the King Abdulaziz University Hospital in Riyadh, Saudi Arabia. Ethical approval was obtained from the local institution's review committee and consent was obtained from all participants. For the purposes of this study, subjects were classified into categories of abnormal glucose homeostasis if they had a single abnormal FPG. 142 patients had type-2 diabetes (FPG > 7.1 mmol/L) (97 males and 45 females). All patients with diabetes were treated with a low-carbohydrate diet, with or without oral antidiabetetic agents, mainly glibenclamide and metformin. 86 pre-diabetic subjects (49 males and 37 females) were defined on the basis of modified ADA criteria [24]. Pre-diabetes was diagnosed when the fasting plasma glucose was between 5.6 mmol/L – 7.0 mmol/L (100–126 mg/dL). 80 Saudi individuals (45 males and 35 females), with no prior history of CHD or diabetes, were recruited as controls (no diabetes).

### Anthropometric parameters

All subjects underwent a full physical examination and completed a general questionnaire. Height was measured to the nearest 0.5 cm and weight to the nearest 0.1 kg. Waist circumference (cm) was measured at the horizontal circumference midway between the lowest rib margin and the iliac crest and the hip circumference (cm) was measured at the maximum circumference over the buttocks. Body mass index (BMI) was calculated as weight (kg) divided by height (m) squared. Groups were matched for BMI.

### Laboratory methods

Blood samples were collected after a 12-hour fast for the determination of glucose, insulin, total cholesterol, HDL, triglycerides, apolipoprotein A1 (apo A1), apolipoprotein A2 (apo A2) and leptin. All samples were stored at -70°C prior to analysis by using routine laboratory methods, except for insulin and leptin assay. Insulin was analyzed by a solid phase enzyme amplified sensitivity immunoassay (Medgenix INS-ELISA, Biosource, Belgium). Leptin concentrations were measured by radio-immunoassay (Linco Research, St. Charles, MO). Homeostasis model assessment-insulin resistance (HOMA-IR) and β-cell function index were derived using the HOMA equation [25].

### Statistical Analysis

Data were analysed using the Statistical Package for the Social Sciences (SPSS) version 10 (SPSS, Evanston, IL, USA) for Windows. Biochemical parameters not normally distributed were analyzed after being logarithmically transformed. Students' unpaired t-test and one-way analysis of variance (ANOVA) were used to compare the results of the different groups. Simple and partial correlation coefficients between the variables were determined and multiple regression analysis was performed to determine the relationships between the variables of interest. Data were expressed as mean (SD) or median (range); statistical significance was set at p < 0.05.

## Results

Table 1 presents the clinical and metabolic characteristics of the male subjects. It is evident that those in the diabetes group were significantly older (p < 0.0001), with higher fasting glucose and leptin levels (p < 0.0001, 0.012 respectively) than those in the pre-diabetes and control groups. At the same time, the pre-diabetes group had significantly elevated systolic blood pressure (p = 0.046) and fasting glucose levels (p < 0.0001), compared to the controls.

**Table 1:** Clinical characteristics and metabolic parameters of male subjects with type-2 diabetes, pre-diabetes and no diabetes

	No Diabetes	Pre-diabetes	Type-2 Diabetes
N	45	49	97
Age (years)	45.7(12.6)	50.1(11.6)	54.5(10.7)^**f**^
Systolic blood pressure (mm Hg)	123.1(16.9)	132.8(25.1)^**a**^	130.7(17.5)
Diastolic blood pressure (mm Hg)	79.9(9.1)	85.1(11.4)	82.9(10.5)
BMI (Kg/m^2^)	29.2(7.3)	28.5(4.3)	27.3(4.1)
Waist (cm)	93.7(18.2)	98.6(10.1)	96.4(15.1)
Hips (cm)	95.9(15.7)	100.5(13.9)	99.8(14.4)
Fasting Glucose (mmol/L)	4.8(0.6)	6.1(0.4)^**f**^	13.7(5.0)^**f**^
Cholesterol (mmol/L)	5.2(1.6)	5.6(1.4)	6.5(1.9)^**f**^
HDL (mmol/L)	0.94(0.3)	1.2(0.4)	0.9(0.4)
Triglyceride (mmol/L)	1.8(0.6–6.7)	1.9(0.7–6.3)	2.5(1.6–15)
LDL (mmol/L)	3.3(1.1)	3.9(1.6)	4.2(1.8)^**b**^
Insulin μmol/L *	12.6(2–99)	12.3 (2–36)	16.2(2–70)
Leptin ng/mL *	3.9(0.8–20)	7.6(1.2–72)^**d**^	12.4(3.2–72)^**c**^
APO A1 mg/dL	0.9(0.3)	0.9(0.5)	0.9(0.3)
APO A2 mg/dL	0.4(0.1)	0.4(0.1)	0.3(0.1)
HOMA-IR *	3.3(0.3–23.8)	3.5(0.5–10.2)	9.6(1.1–49.7)^**e**^

Data are presented as means (SD) or as medians (interquartile range) for insulin, leptin and HOMA-IR data. The P-values shown are comparisons of metabolic characteristics for case control versus either prediabetic patients or those with T2DM: a) 0.046; b) p = 0.03; c) p = 0.012; d) p = 0.004; e) p = 0.001; f) p < 0.0001.

Table 2 highlights the clinical and metabolic parameters in the females. In the diabetes and pre-diabetes group, FPG and LDL were significantly higher than in the control. Total cholesterol was also significantly higher in women with diabetes (p = 0.03).

**Table 2:** Clinical characteristics and metabolic parameters of female subjects with type-2 diabetes, pre-diabetes and healthy control subjects

	No diabetes	Pre-diabetes	Type-2 diabetes
N	35	37	45
Age (years)	43.6(11.3)	43.3(11.7)	49.0(12.6)^**d**^
Systolic blood pressure (mm Hg)	122.8(23.1)	127.3(24.2)	123.6(18.6)
Diastolic blood pressure (mm Hg)	79.4(16.5)	79.8(8.03)	80.7(10.3)
BMI (kg/m^2^)	30.4(6.4)	32.5(8.4)	32.5(10.3)
Waist (cm)	90.5(12.5)	91.8(13.5)	96.2(17.9)
Hips (cm)	105.3(14.1)	104.8(17.9)	106.7(20.8)
Fasting Glucose (mmol/L)	4.8(0.9)	6.1(0.4)^**b**^	14.1(5.5)^**b**^
Cholesterol (mmol/L)	5.2(1.3)	5.3(1.1)	6.6(1.9)^**b**^
HDL (mmol/L)	1.1(0.4)	0.9(0.4)	1.1(0.3)
Triglyceride (mmol/L)	1.9(1.9)	1.6(0.9)	2.6(1.4)
LDL (mmol/L)	3.5(1.4)	3.6(1.1)^**d**^	4.4(1.5)^**c**^
Insulin (μmol/L)*	15.2(2–62)	13.5(6–26)	14.04(1–52)
Leptin ng/mL *	10.2(0.25–34.8)	14.09(2.8–44.4)^**a**^	13.3(3.6–49.1)
APO A1 mg/dL	0.8(0.4)	0.9(0.4)	1.2(2.5)
APO A11 mg/dL	0.4(0.1)	0.4(0.1)	0.4(0.2)
HOMA IR *	3.1(0.3–12.5)	3.6(1.5–7.3)	7.8(0.6–28.4)^**b**^

*Data are presented as means (SD) or as medians (interquartile range) for insulin, leptin and HOMA-IR data. The P-values shown are comparisons of metabolic characteristics for case control versus either prediabetics or patients with T2DM: a) 0.046; b) p = 0.03; c) p = 0.004; d) p < 0.001

Serum leptin was significantly higher in the men with diabetes than in their non-diabetic counterparts (12.4 [3.2–72]; p = 0.0001). In the female patients, serum leptin was higher in those with pre-diabetes (14.09 [2.8–44.4]) than in the controls.

**Figure 1:**
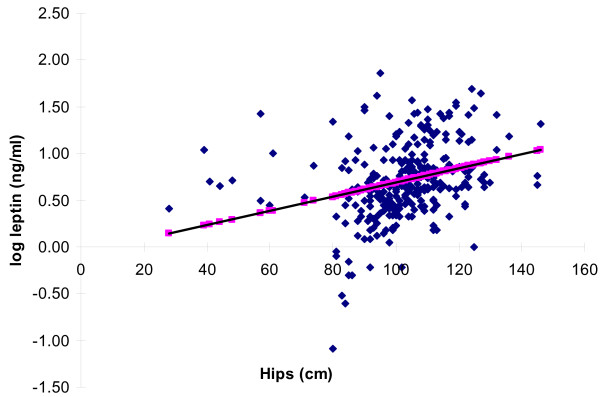
**Log leptin (ng/ml) vs. Hip circumference (cm)**. The correlation of log leptin (ng/mL) with hip circumference (cm) using univariate analysis across all Saudi subjects, case controls, T2DM and pre-diabetic subjects. Blue denotes the actual values with the line of best fit shown in square (R^2 ^0.086 p-value = 0.006).

Stepwise linear regression analysis revealed a positive correlation between log leptin and hip circumference and BMI (R = 0.31, p = 0.002; R = 0.26, p = 0.006 respectively). Even after adjustment for BMI, there was a positive association between log leptin and hip circumference with an R^2 ^= 0.086 and p value of 0.006 (see Figure 1). The rest of the findings were non-contributory.

## Discussion

Insulin resistance is the common factor in a range of risk factors for atherosclerosis, specifically hypertension, dyslipidemia and abnormal glucose metabolism. As has been shown in other populations [26-29], the pre-diabetic males had a significantly higher systolic blood pressure and fasting plasma glucose than the control subjects had. Many [30,31], but not all studies [32,33] of the general population have shown a positive association of indices of insulin resistance with arterial wall changes, CHD and CVD. The clustering of risk factors increases the risk of atherosclerosis [34-36].

Data were presented according to gender, since it is already an established fact that leptin levels are significantly higher in women than in men. There are several possible explanations for the difference. One is that females have more adipose tissue than males, but a growing literature indicates that estrogen, especially at higher levels, will stimulate the production of leptin, whereas androgens will suppress the levels of leptin [37]. In this cross-sectional study, we found positive correlations between leptin and hip circumference in pre-diabetic patients. This is consistent with a previous study, which also showed that this group is at high risk of developing CHD [38]. The positive association with hip circumference remained significant after adjusting for gender and BMI, which confirmed the results in previous studies for healthy men and women [39,40]. With impaired glucose tolerance, small changes in circulating insulin also alter insulin levels [41].

Serum leptin concentration may contribute to the risk of CVD by altering lipid metabolism and contributing to hypertension via the activation of the sympathetic nervous system and increasing renal sodium re-absorption [42,43]. However, in our study there was no correlation between leptin and blood pressure. In another study, leptin signaling directly promoted atherosclerosis and may therefore represent a therapeutic target for the prevention of atherosclerosis [44]. Lichnovska and her colleagues in their recent report mentioned the significant role of serum leptin in the progression of insulin resistance, but this was not confirmed in the present study [45]. The difference can be due to the fact that the earlier study considered elderly and hyperlipemic patients, while the present study focused on pre-diabetic, diabetic and non-diabetic adults. Our results, however, confirm the results of Adamia et al., which reveal no correlation between leptin and insulin resistance [46].

A family history of diabetes was found to have a negative association with leptin in pre-diabetic patients; this confirms a previous study which reported that healthy lean non-diabetic Asian Indians are more insulin resistant than other ethnic groups, despite similarities in their living environment [47]. Waist circumference has been postulated to have closer associations with the biomarkers of CHD than BMI has [48]. The association of leptin to waist circumference (a surrogate for upper body obesity) and hip circumference confirms findings in Mexican American populations [49,50]. After adjustment for BMI, serum leptin concentrations in pre-diabetic men were independent of waist circumference, but in women they were associated with hip circumference. Hip circumference is a proxy measure of peripheral fat in serum leptin concentrations, aside from the fact that women have a significantly larger volume of subcutaneous fat than do men. In the control subjects, leptin concentrations were directly and significantly related to subcutaneous fat, with a strong inverse relationship to waist-hip ratio. When the results were considered according to gender, it was found that 33.1% of the male Saudis with waist circumference >102 cm, had diabetes. In female subjects, 27% of those with a high waist circumference ≥88 cm had diabetes, compared to only 13.4% of those with a normal waist circumference [20]. Leptin is related to waist and hip circumference and is directly proportional to body fat. However, in those with diabetes, this relationship is lost, reflecting the effect of other factors, including hyperinsulinaemia and the activation of sub-clinical inflammation.

## Conclusion

The significant correlation of leptin to selected anthropometric measurements of obesity is confirmed in non-diabetic Saudi Subjects. In those with diabetes, this relationship is lost, reflecting the effect of other factors, including hyperinsulinaemia and the activation of sub-clinical inflammation.

## Competing interests

The author(s) declare that they have no competing interests.

## Authors' contributions

NA for the concept and design as well as statistical analysis; OA for the drafting and revising of the manuscript; KA and AJ for the acquisition and interpretation of data; MM and MK for the screening and collection of data; KS for the concept and final revision of the manuscript. All the authors have read and approved the final version.
